# Applying Machine Learning of Erythrocytes Dynamic Antigens Store in Medicine

**DOI:** 10.3389/fmolb.2019.00019

**Published:** 2019-04-03

**Authors:** Mahmoud Rafea, Passant Elkafrawy, Mohammed M. Nasef, Rasha Elnemr, Amani Tariq Jamal

**Affiliations:** ^1^Central Lab of Agriculture Expert Systems, Giza, Egypt; ^2^Mathematics and Computer Science Department, Faculty of Science, Menoufia University, Shibin El Kom, Egypt; ^3^Computer Science Department, Faculty of Computing and Information Technology, King Abdulaziz University, Jeddah, Saudi Arabia

**Keywords:** mass spectrometry, disorders diagnosis, erythrocytes dynamic antigens store (EDAS), biomarkers, computer tools in clinics, mathematical model

## Abstract

Erythrocytes Dynamic Antigens Store (EDAS) is a new discovery. EDAS consists of self-antigens and foreign (non-self) antigens. In patients with infectious diseases or malignancies, antigens of infection microorganism or malignant tumor exist in EDAS. Storing EDAS of normal individuals and patients in a database has, at least, two benefits. First, EDAS can be mined to determine biomarkers representing diseases which can enable researchers to develop a new line of laboratory diagnostic tests and vaccines. Second, EDAS can be queried, directly, to reach a precise diagnosis without the need to do many laboratory tests. The target is to find the minimum set of proteins that can be used as biomarkers for a particular disease. A hypothetical EDAS is created. Hundred-thousand records are randomly generated. The mathematical model of hypothetical EDAS together with the proposed techniques for biomarker discovery and direct diagnosis are described. The different possibilities that may occur in reality are experimented. Biomarkers' proteins are identified for pathogens and malignancies, which can be used to diagnose conditions that are difficult to diagnose. The presented tool can be used in clinical laboratories to diagnose disease disorders.

## Introduction

The main purpose of proteomics-science is to identify and characterize protein expression in biological systems. Proteomics is an extremely large field consisting of a different collection of platforms. Mass spectrometry (MS) technology is an essential device in these platforms. MS has a powerful use for protein identification and profiling experiments (Barnes and Gray, 2003; Pasini et al., 2010; Timms et al., 2016; Wang et al., 2016; Bryk and Wisniewski, 2017).

Proteomics methods which are based on MS hold special promise for the discovery of novel biomarkers that might form the foundation for new clinical tests. Advances in methods and technology now enable construction of a comprehensive biomarker pipeline from five essential process components: candidate discovery, quantification, verification, research assay optimization, and biomarker validation (Rifai et al., 2006).

**Figure 1:**
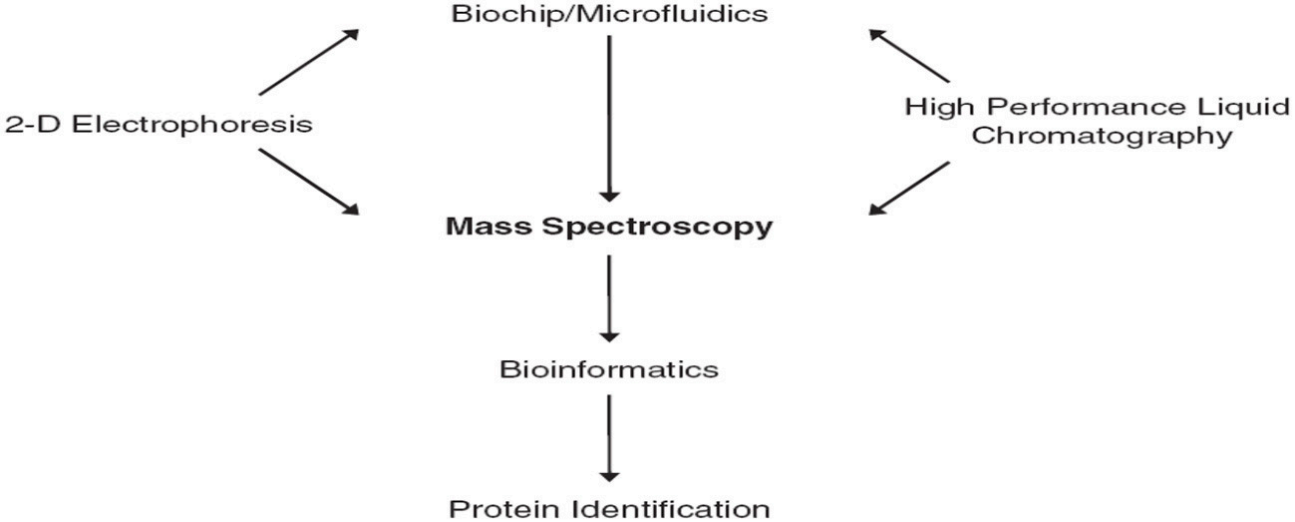
The central role of mass spectrometry in proteomics (Jain, 2010).

Biomarkers discovery depends on the comparison of different physiological states, phenotypes done during controlling (diseased) patient groups. Biomarker discovery using MS techniques requires sensitivity, mass accuracy, and reproducibility. The central role of mass spectrometry in proteomics is shown in Figure 1 (Jain, 2010).

There are many definitions of biomarker (Naylor, 2003)^1^^,^^2^. Meanwhile, we will state the definition of the National Cancer Institute which defines the biomarker as “a biological molecule found in blood, other body fluids, or tissues that is a sign of a normal or abnormal process, or of a condition or disease^3^.”

One of the most important applications of specific biomarkers is to find the tumor at an early stage even before clinical symptoms are developed. Early detection of cancer would benefit patients; as more tumors should be treated more efficiently (Borrebaeck, 2017). This would certainly increase overall survival. The World Health Organization (WHO) proposed that millions of cancer patients could be saved from premature death if early detection and treatment were available (World Health Organization, 2007).

Apart from early diagnosis, biomarkers could also provide physicians with actionable information leading to the evidence-based selection of the optimal therapy (predictive biomarkers) and improved and more precise prognostication of disease progression (prognostic biomarkers)^4^. Ideally, protein biomarkers should be found in a minimally invasive liquid biopsy, such as a simple blood sample. However, the question is whether blood contains enough information and whether we are even close to this scenario? Tremendous efforts have been made over recent decades to find protein cancer biomarkers of clinical utility (Brennan et al., 2010; Neagu et al., 2011; Vlahou, 2013; Franzi et al., 2014).

There is over a thousand single candidate cancer biomarkers have been known for several years (Polanski and Anderson, 2007). However, the US Food and Drug Administration (FDA) approved that none of these is routinely used for early clinical diagnosis, except a few of them for example, CA125 (also known as mucin 16) for ovarian cancer, prostate-specific antigen (PSA) for prostate cancer and CA19-9 for pancreatic cancer have been proposed to be useful for longitudinal disease monitoring (Füzey et al., 2013; Pavlou et al., 2013; Menon et al., 2015).

This work moves from single biomarker to multiple biomarkers. Multiple biomarkers can provide significantly increased diagnostic accuracy. Combinations of biomarkers contain much more information than a single biomarker, where the latter does not display sufficient discriminatory power to substantially affect clinical decisions (Borrebaeck, 2017).

**Figure 2:**
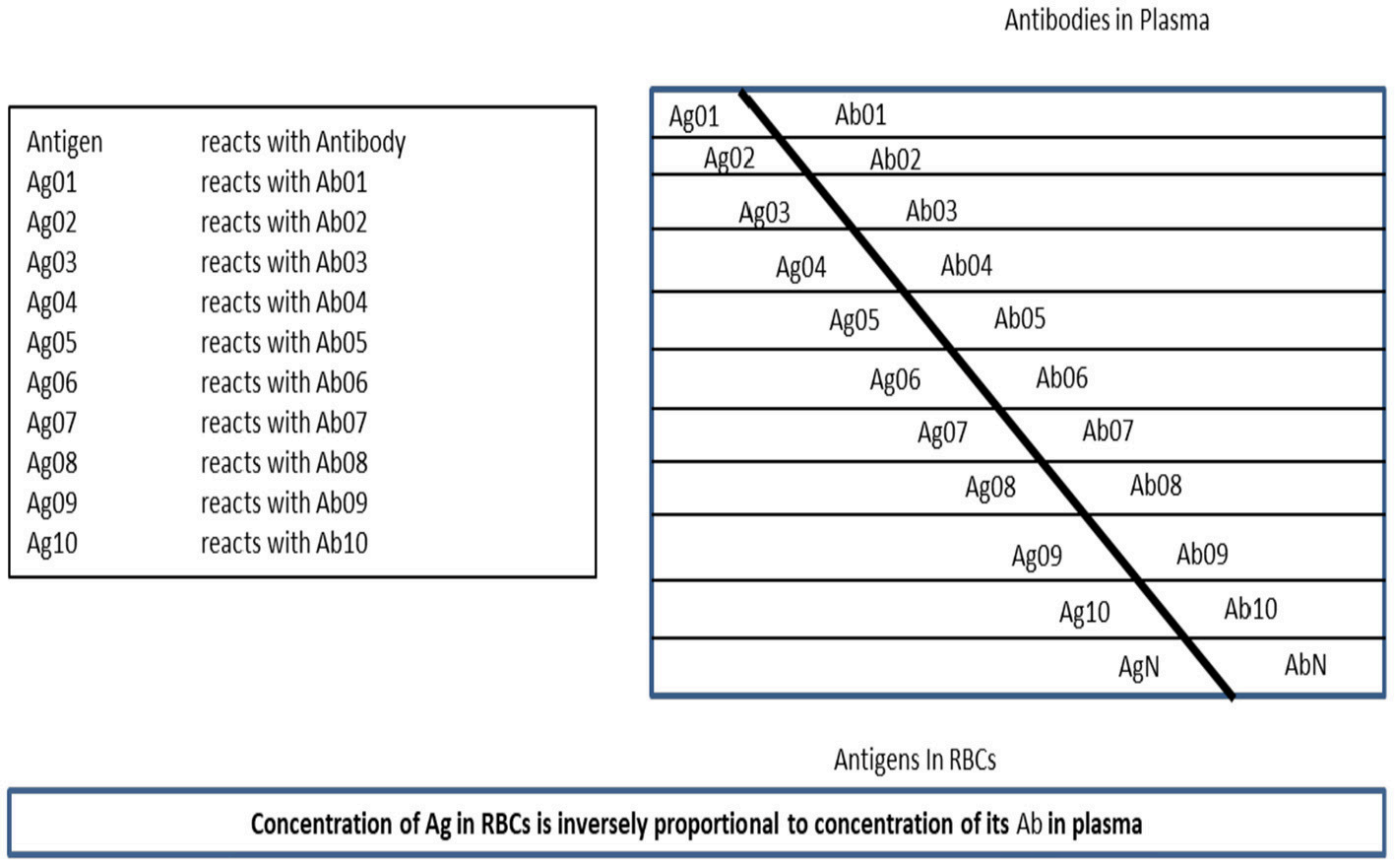
The relation between plasma antibodies and EDAS.

Rafea and Souchelnytskyi (2012) observed and described a phenomenon related to the protein content of the Red Blood Cell (RBC). It was noticed that the plasma contains antibodies against some of RBC proteins, which are contained within RBC cytoplasm of the same person. Many experiments are done to understand the relation between RBC antigens content and their relation to plasma antibodies. Those experiments conclude that the antigens exist in the RBC cytoplasm have relation to immune tolerance and that RBC has a dynamic store of: body antigens [Tissue Specific Antigens (TSA)], food antigens, environment antigens, bacterial commensals antigens, and disease antigens whether microbial, viral, or tumors. They named this store: Erythrocytes Dynamic Antigens Store (EDAS). Figure 2, depicts the relationship between EDAS and plasma antibodies.

The first application or invention which is based on EDAS is named TB-KIT (PCT/EG/000013, 2017). TB-KIT is Lateral Flow Chromatographic Assay (LFCA) for determining the antigens concentration of Mycobacterium tuberculosis complex, in the cytoplasm of blood erythrocytes (hemolysate) (PCT/IB/054691, 2007; WO/059112, 2012). The test has been verified and validated. It is currently available and in the process of certification.

A random generation of EDAS was described in Rafea et al. (2010) and Rafea and Souchelnytskyi (2012). Meanwhile, this generation of the EDAS model was very simple and did not reflect the real EDAS. It was based on classifying proteins into normal and abnormal, only, without specifying the nature of these proteins.

As a matter of fact, identifying proteins of RBC that reacts with self-antibodies and storing the identity of those proteins in a database for different diseases disorders and normal individuals will help in many directions. The first aim is to efficiently diagnose serious disease conditions as early as possible. This helps to monitor the treatment of these diseases conditions. Hence, in Rafea et al. (2010) and Rafea and Souchelnytskyi (2012) they proposed a technique to discover biomarkers of diseases based on EDAS. However, they did not show which disease or set of diseases can be applied? They used one category of diseases. Also, they did not make any experiment to verify the model. In fact, classifying the antigens within the EDAS record will help in many other directions which will be the subject of other research articles.

The main challenge of our research is its ability in diagnosing the disease at deep immunological levels. In effect, it will help to accurately diagnose conditions that are difficult to diagnose. This research is based on a new mathematical model of EDAS to simulate reality. So that the biomarkers discovery technique is developed using supervised machine learning algorithms. The training datasets of bio-samples created hypothetically in the database. The developed biomarkers discovery technique described identifying a set of biomarkers of each disease. The work is done for two categories of diseases; pathogens and malignancies.

In the real world, the EDAS is identified in laboratories through four steps. First, prepare affinity column chromatography using proteins G and/or A. Second, add patient plasma to the column which binds immunoglobulins (IgG). Third, add patient erythrocytes hemolysate so that IgGs, which act as a ligand, bind antigens representing EDAS. Last, elute the column and collect EDAS proteins. The separation of EDAS is followed by the identification of its proteins content using LC/MS/MS (Pasini et al., 2010; Bryk and Wisniewski, 2017).

The developed mathematical model for EDAS is described in more details in section 2. The developed biomarker discovery technique based on the EDAS store is described in section 3. The diagnostic model is described in section 4. Experiments are described in section 5. Results and discussion are described in section 6. Conclusion and future directions are explained in section 7.

## Mathematical Description of EDAS

The mathematical description will include the mathematical definition followed by the generation of hypothetical EDAS domain.

### Mathematical Definition

1. The set E = {e_i_, e_i+1_, …, e_n_} where e_i_ is a protein from EDAS and belongs to the individual surrounding environment, e.g., mosquito protein, where 1 ≤ i ≤ n.
2. The set E′ ⊂ E, there exit EDAS where E′ = E ∩ EDAS.
3. The set F = {f_i_, f_i+1_, …, f_n_} where f_i_ is a protein from EDAS and belongs to an individual's food, where 1 ≤ i ≤ n.
4. The set F′ ⊂ F, there exit EDAS where F′ = F ∩ EDAS.
5. The set C = {c_i_, c_i+1_,…, c_n_} where c_i_ is a protein from EDAS and belongs to bacterial commensals, where 1 ≤ i ≤ n.
6. The set C′ ⊂ C and there exit EDAS where C′ = C ∩ EDAS.
7. The set T = {t_i_, t_i+1_, …, t_m_} where t_i_ is a protein from EDAS and is a Tissue-Specific Antigen, where 1 ≤ i ≤ m.
8. The set T′ ⊂ T, there exist EDAS where T′ = T ∩ EDAS.
9. The set G = {Gi, Gi+1, …, Gk} where Gi is a pathogen that can induce a disease, where 1 ≤ i ≤ k.
10. The set Gi ={g_ij_, g_ij+1_, …, g_iq_} where g_ij_ is a protein in the proteome of Gi, where 1≤ j ≤ q.
11. The set ${\text{G}}_{\text{i}}^{\prime }$ ⊂ Gi and there exist EDAS where ${\text{G}}_{\text{i}}^{\prime }$ = Gi ∩ EDAS.
12. The set M = {Mi, Mi+1, …, Mk} where Mi is a malignant tumor, where 1≤ i ≤ k.
13. The set Mi ={m_ij_, m_ij+1_, …, m_iq_} where m_ij_ is a protein in the proteome of Mi, where 1≤ j ≤ q.
14. The set ${\text{M}}_{\text{i}}^{\prime }$ ⊂ M_i_ and there exist EDAS where ${\text{M}}_{\text{i}}^{\prime }$ = Mi ∩ EDAS.
15. The set HD = {hd_i_, hd_i+1_, …, hd_r_} where hd_i_ is a hypothetical EDAS, where 1 ≤ i ≤ r.
16. The set hd_i_ = E′ ∪ F′ ∪ C′ ∪ T ′ ∪ ${\text{G}}_{\text{i}}^{\prime }$ ∪ ${\text{M}}_{\text{i}}^{\prime }$.

### The Generation of Hypothetical EDAS Domain

The EDAS domain is defined in the previous section as HD. A patient EDAS: hd_i_ is created according to the following parameters and procedures:

- Initially, the parameters:
  1. The number of elements (n) in E (environmental proteins) is 3000 protein.
  2. The number of elements (n) in F (food proteins) is 3000 protein.
  3. The number of elements (n) in C (commensal bacterial proteins) is 3000 protein.
  4. The number of elements (m) in T (tissue-antigens) is 10,000 protein.
  5. The number of pathogens (k) in (G) is 20 pathogen.
  6. The number of proteins (q) for each pathogen (Gi) is 500 protein.
  7. The number of malignancies (k) in (M) is 20 malignancy.
  8. The number of proteins (q) for each malignancy (Mi) is 500 protein.
- Consequently, each patient hd_i_ is generated through the following steps:
  1. The random generation of environment proteins: set E′ which has a number of elements (RE) generated randomly using a Normal distribution from the set E.
  2. The random generation of food proteins: set F′ has a number of elements (RF) generated randomly using a Normal distribution from the set F.
  3. The random generation of commensal bacterial proteins: set C′ which has a number of elements (RC) generated randomly using a Normal distribution from the set C.
  4. The random generation of Tissue-Specific Antigens: set T′ which has a number of elements (RT) generated randomly using a Normal distribution from the set T.
  5. The random generation of a pathogen or malignant tumor. First, a random flag is generated that has a value between 0 and 2.
    - If flag = 0, there will be neither pathogen nor malignant tumor proteins in hd_i_.
    - If flag = 1, then hd_i_ will have pathogen proteins.
      - A pathogen “Gi” is selected randomly from the set G.
      - The random generation of pathogen proteins: subset ${\text{G}}_{\text{i}}^{\prime }$ has a number of elements (RGi) generated randomly using a Normal distribution from the set Gi.
    - If flag = 2, then hd_i_ will have malignant tumor proteins.
      - A malignant tumor “Mi” is selected randomly from the set M.
      - The random generation of malignant proteins: subset M_i_' has a number of elements (RMi) generated randomly using a Normal distribution from the set Mi.

## Materials and Methods

The importance of this work is based on the fact that one can diagnose precisely disease conditions that are difficult to diagnose from a set of possible diseases using a single sample and a single test. In this paper, the algorithms, which are documented in (PCT/EG/000013, 2017), are modified to include different categories of diseases; namely: pathogens and malignancies.

### Biomarker Discovery Tasks

The main task is to discover a unique protein(s) associated with a particular disease. Usually, we will find more than one protein. Consequently, any of the unique proteins can be selected and used as a biomarker in the diagnostic process and/or treatment monitoring. However, to achieve a more accurate diagnosis a set of biomarkers (proteins) can be used. Interestingly, the use of unique protein(s) associated with a particular disease can be used to develop a vaccine, a point that needs medical research. Disease biomarkers are discovered from the RBC by knowing the normal proteins. Normal proteins are discovered first in order to differentiate them from the diseased ones. The biomarker discovery algorithms are done in two main steps.

**Step 1: Normal protein (P′ normal) extraction**
  Algorithm 1 shows the developed pseudocode of this step. Firstly, collect the proteins (P normal) from patient records that are diagnosed as normal (Normal Cases); then filter the set (P normal) to exclude the proteins which have sharing occurrence <5% in the records of normal cases. Those proteins are excluded because their low occurrence may indicate a biological error. In effect, those abnormal proteins are not related to a particular disease. In some sense, this is taken into consideration to mimic nature which is almost 95% perfect. The remained (retained) proteins are considered as Normal Proteins (P′ normal).
**Step 2: Disease biomarkers (P′dj) extraction**
  Biomarker(s) is/are protein(s) which exist(s) in all patients' records having the same diagnosis. Firstly, we detect common-shared proteins for each disease (Pdj). Then we remove the set of normal proteins (P′ normal) that exist in the common-shared proteins (Pdj) for each disease (dj) separately as in the equation (P′dj = Pdj– P′ normal).
  First: Detecting common-shared proteins for a particular disease (Pdj)
  The main goal of this step is to detect the common-shared proteins for each disease while using pathogen and malignant tumor diseases. From patients' records which are stored in the database, we can select all records for each disease (dj) separately. Then the set of all common-shared proteins in those records is constructed (Pdj). Algorithm 2 shows the developed pseudocode of this step.
  Second: Discovering biomarkers′ proteins (P′dj)
  In the last step of the biomarkers detection stage, we attempt to discover biomarkers' proteins for more than one category of diseases. This step should exclude the set of normal proteins (P′ normal) that exist in common-shared proteins (Pdj) for each disease (dj) separately. This excluding is done by differentiating the common-shared proteins from the set of normal proteins (P′ normal–Pdj) to get (P′dj). The result of each disease (dj) (pathogen and malignant tumor) is a minimum set of proteins that can be used as biomarkers for this disease. Algorithm 3 shows the developed pseudocode of this step.

**Algorithm 1:** Detecting the Normal Proteins

#Input: normalCases be the list of all Normal Cases
#Output: normalProteinsbe the list of Normal proteins collected with occurrence > 5% (P′ normal)
# the union of normal cases to get a single occurrence of each protein in a list
Initialize collectedProteins as union of all proteins in normalCases
Initialize normalProteinsas empty list
noCases = length (normalCases)
for each protein incollectedProteins,
if (protein in normalProteins)
incrProteinCounter(protein)
else
add protein to normalProteins
createProteinCounter(protein)
end if
end for
#filter collectedProteinsfrom low occurring proteins <5%
for each protein incollectedProteins
pPercent = getProteinCounter(protein) ^*^ 100 / noCases
if (pPercent <= 5)
remove protein from normalProteins
end if
end for
end algorithm 1

**Algorithm 2:** Detecting the common-Shared Proteins of Each Disease

#Input: diseasesList be the list of all Diseases
#Input: patientList be the list of all patients' records
#Output: commonDiseasesProteins be the list of all common-shared disease proteins (Pd_*j*_*)*
Initialize commonDiseasesProteins as empty lists with length of diseasesList
Initialize allProteins as empty list
for each Disease in diseasesList
Initialize commonDiseasesProteins[Disease] empty list
diseaseRec = select all patient records of Disease
dr = first record in diseaseRec
# find proteins that exist in all records
foreachdisProtein in dr
flag = true
foreach rec indiseaseRec
ifdisProtein does not exist in rec
flag = false
endforeach
if (flag) add disProteintocommonDiseases
Proteins[Disease]
end foreach
endfor
return commonDiseaseProteins
end algorithm 2

**Algorithm 3:** Detecting the Biomarkers' Proteins

#Input: normalProteins be the list of all Normal Proteins (P′ normal)
#Input: commonDiseasesProtein be the list of common proteins of each Disease (Pd_j_)
#Input: diseasesList be the list of all Diseases
#Output: biomarkersList (P′d_j_)
Initialize biomarkersList as empty lists with length of diseasesList
for each Disease
foreachdisProteinin commonDiseasesProteins[Disease]
if disProtein does not exist in the normalProteins
add to biomarkersList [Disease]
end foreach
endfor
return biomarkersList
end algorithm 3

## The Diagnostic Model

In this section, we verify the consistency of the model through generating a new case and testing how it can match with the cases in the database. As described in the following mathematical expression;

**Table:** 

*∀ Dis in DiseasesSet*	
*if BiomarkerSet of Dis ∩ NewCase*	
*≠ Null then Diagnosis = Dis*	(1)
*elseifDiseaseProteins of Dis ∩ NewCase*	
*≠ Null then Diagnosis = Dis*	(2)
*else NewCase is Normal*	

In the first situation “Equation (1),” the integration is straightforward; if the BiomarkerSet is a subset of the new patient case where the intersection between the BiomarkerSet and the new patient case gives a result not null. Then this patient suffers from a corresponding disease (*Dis)*.

If the BiomarkerSet is not a subset of the new patient case, and the intersection between the BiomarkerSet and the new patient is null, then the case cannot be directly integrated into the database. In this situation, Equation (2) is executed. If intersecting the already known diseases' proteins set (not only the biomarkers proteins) with the new patient case is null, this indicates that this patient is normal. However, if the intersection is not null this indicates that this patient is suffering from the corresponding disease (*Dis)*. In effect, this indicates that the biomarker set is incomplete. Consequently, the tool updates the BiomarkerSet by re-executing the module of discovering biomarker of disease (*Dis*).

## Experiments

**Figure 3:**
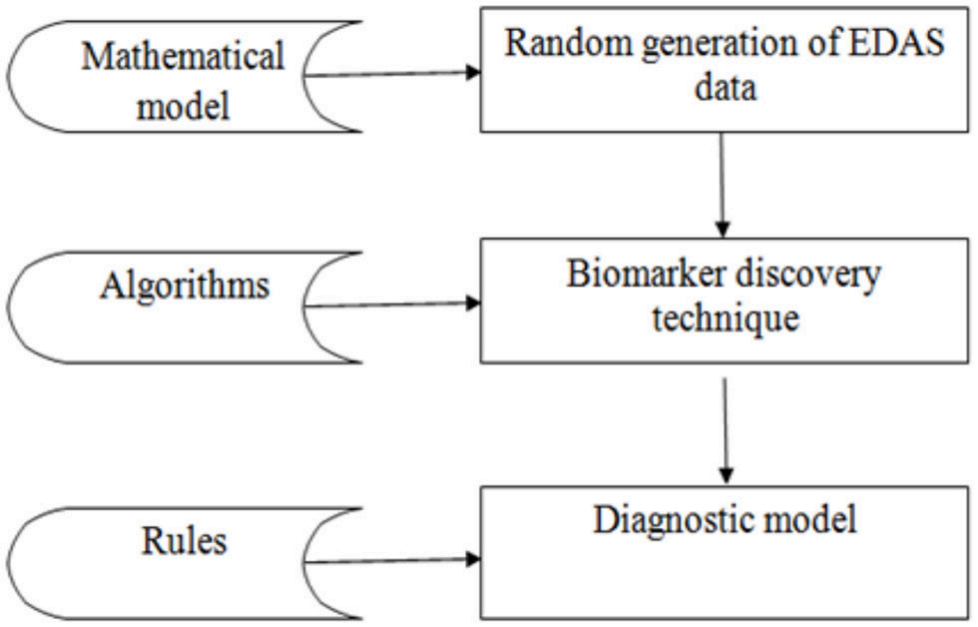
Workflow pipeline of the experiment.

The experiment is divided into three phases as shown in Figure 3: the random generation of the EDAS data, the biomarkers discovery phase, and the diagnostic phase. The data phase is based on generating records for 100,000 cases. Each case is generated randomly from the set of proteins as described in the mathematical model in section Mathematical Description of EDAS. Then the proposed biomarker discovery technique is applied in these cases.

The experiment is performed on MacBook Pro, 2.9 GHz Intel Core i5 and 8 GB of RAM, the database is created in Microsoft SQL Server 2008, the algorithms are implemented in C#.

***Phase 1***: Random generation of EDAS data

In this step, the artificial dataset of proteins is generated randomly based on Normal distribution and according to the previous mathematical model.

Firstly, a pool of normal proteins is constructed, from the following categories:

1. 3,000 environment proteins (P1, …., P3000)
2. 3,000 food proteins (P3001, …., P6000)
3. 3,000 bacterial commensal proteins (P6001, …., P9000)
4. 10,000 tissue proteins (P9001, …., P19000).

From this pool, the set of normal proteins (N) for each case is created randomly as the following:

1. E′ is composed randomly from the set (E) using (RE). The arity (RE) is randomly generated, where RE ≤ 3000 proteins.
2. F′ is composed randomly from the set (F) using (RF). The arity (RF) is randomly generated, where RF ≤ 3,000 proteins.
3. C′ is composed randomly from the set (C) using (RC). The arity (RC) is randomly generated, where RC ≤ 3,000 proteins.
4. T′ is composed randomly from the set (T) using (RT). The arity (RT) is randomly generated, where RT ≤ 10,000 proteins.

The union of these sets (E′, F′, C′, T′) form the set of normal proteins (N).

Secondly, a pool of pathogens proteins is generated like the following:

- 20 types of pathogens (G1, …., G20). Each one of them is composed of N + Gi′. Gi′ is composed randomly from the set (Gi) using (RGi). The arity (RGi) is randomly generated, where RGi ≤ 500 proteins. This ensures the uniqueness of the biomarkers.

Thirdly, a pool of malignancies proteins is generated like the following:

- 20 types of malignancies (M1, …., M20). Each one of them is composed of N + Mi′. Mi′ is composed randomly from the set (Mi) using (RMi). The arity (RMi) is randomly generated, where RMi ≤ 500 proteins. This ensures the uniqueness of the biomarkers.

Lastly, 100,000 transactions are created randomly as the following:

A random function is operated to specify if the record is a normal case, a pathogen case, or a malignancy case. In the normal case, the set of proteins is generated randomly only from the pool of normal proteins. In the pathogen case, the set of proteins is generated randomly from the pool of normal proteins and the pool of pathogens proteins. In the malignancy case, the set of proteins is generated randomly from the pool of normal proteins and the pool of malignancies proteins.

***Phase 2*:** Applying the biomarker discovery technique on the previously generated data in phase 1. The aim is to detect a set of biomarkers for each disease separately from the randomly generated records.

***Phase 3*:** Applying the diagnostic model to the new generated case. The aim is to diagnose a new case. Queries are done to verify the diagnosis. Firstly, we generate a new case as described in phase one. This new case is documented in the XML file. Secondly, we can select this XML file. Thirdly, the case can be evaluated. This is by comparing the set of proteins for this case with the already known sets of biomarkers for all diseases disease by disease. Lastly, we can save this new case with its diagnosis.

## Results and Discussion

In the experiment, there are 100,000 patients' records stored in a database. Where:

1. The number of normal cases is 30,719 records.
2. The numbers of patients who have pathogens are 27,539 records.
3. The numbers of patients who have malignant tumors are 41,742 records.

Tables 1, 2 shows the quantitative results.

**Table 1:** Results of the experiment for pathogens.

**Disease**	**Number of records**	**Number of biomarker proteins**
G1	1,371	31
G2	1,303	42
G3	1,346	25
G4	1,310	8
G5	1,390	41
G6	1,365	13
G7	1,395	55
G8	1,396	79
G9	1,399	6
G10	1,319	63
G11	1,346	16
G12	1,420	32
G13	1,404	55
G14	1,403	35
G15	1,407	24
G16	1,351	33
G17	1,333	17
G18	1,438	46
G19	1,403	10
G20	1,440	16

**Table 2:** Results of the experiment for malignant tumors.

**Disease**	**Number of records**	**Number of biomarker proteins**
M1	2,063	30
M2	2,109	43
M3	2,083	30
M4	2,053	19
M5	2,035	35
M6	2,094	24
M7	2,062	116
M8	2,135	13
M9	1,982	23
M10	2,096	21
M11	2,040	29
M12	2,084	37
M13	2,076	28
M14	2,149	32
M15	2,130	11
M16	2,115	32
M17	2,059	85
M18	2,080	50
M19	2,116	26
M20	2,181	41

As shown in Table 1, the number of patients that suffered from the disease (G1) was 1,371. After applying the proposed algorithm, we observed that the number of biomarkers for this disease is 31 proteins. In the case of disease (G15), the number of patients that suffered from this disease was 1,407. After applying the proposed algorithm, we observed that the number of biomarkers for this disease is 24 proteins.

As shown in Table 2, the number of patients that suffered from the disease (M2) was 2,109. After applying the proposed algorithm, we observed that the number of biomarkers for this disease is 43 proteins. In the disease (M18), the number of patients that suffered from this disease was 2,080. After applying the proposed algorithm, we observed that the number of biomarkers for this disease is 50 proteins.

**Figure 4:**
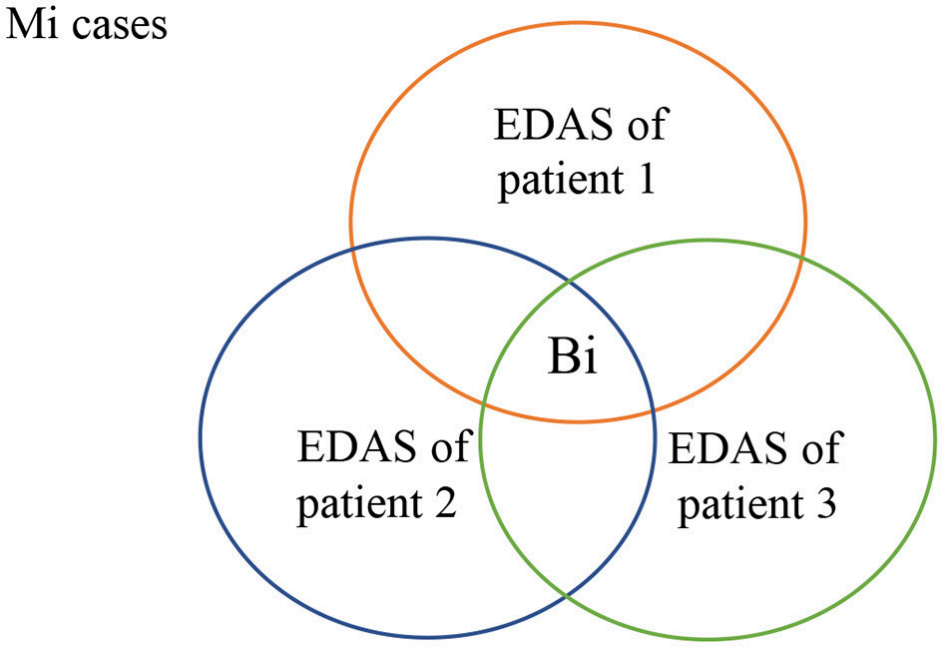
Common-shared malignancy proteins.

As shown in Figure 4, all patients who suffer from malignancy Mi have EDAS consists of N+Mi' proteins, where N represents the normal proteins, and Mi' represents the malignancy proteins. The intersection of all Mi cases after subtracting N from their EDAS, produces a subset of common shared malignancy proteins, B_i_. In other words; B_i_ is a set of biomarkers profiling malignancy Mi.

Each set of biomarkers is unique for a particular disease because the biomarker uniqueness is inherent during disease proteins generation. Obviously, diseases have a lot of proteins that may be shared between diseases. However, those proteins are not considered. Because of they are shared with commensal, environment, food, and tissue proteins. We consider the proteins that are specific for a particular disease.

The results of phase 3 are shown in Table 3. The results contain some patients and some details about their health state such as the patient number, the number of proteins (EDAS), the disease infects for the patient (if found), the number of the biomarkers of this disease, the number of the biomarkers of this disease which found in the set of proteins (EDAS) of the patient, and the Jaccard similarity analysis.

**Table 3:** The results of patients after diagnosis.

**Patient number**	**Patient**	**Patient**	**Patient**	**Patient**	**Patient**
	**no. 1**	**no. 2**	**no. 3**	**no. 4**	**no. 5**
Edas no.	1,958	1,888	1,939	2,069	2,010
Disease	M10	G6	Normal	G18	M8
Number of biomarkers	21	13	Null	46	13
Number of biomarkers found	14	2	Null	45	2
Jaccard similarity	66.67%	15.38%	Null	97.83%	15.38%

**Figure 5:**
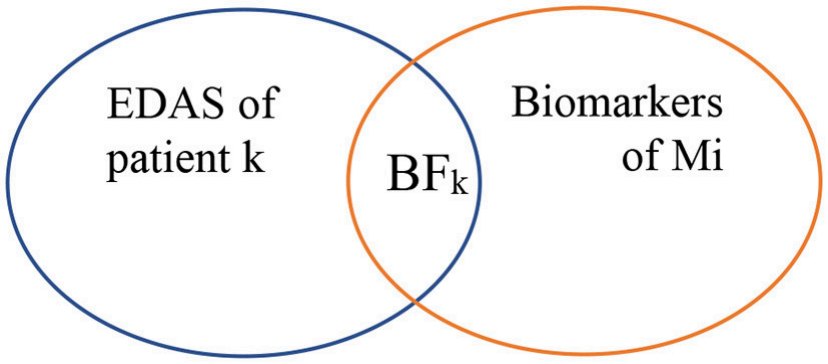
Biomarkers found from EDAS.

As shown in Figure 5, biomarkers of Mi represent the set of biomarkers for a particular malignancy Mi. BF represents the biomarker found at the EDAS of patient j. The intersected set between the set of biomarkers for a malignancy Mi and the EDAS of patient j is considered as BF.

From this point, the Jaccard similarity can be calculated as shown in Table 3. The Jaccard similarity (coefficient) (Fletcher and Islam, 2018) is a term coined by Paul Jaccard to measure similarities between sets. It is defined as the size of the intersection divided by the size of the union of two sets. The Jaccard similarity of

**Table:** 

**Cases**	**Sets**	**Jaccard similarity (%)**
Patient 1	14/21	66.67
Patient 2	2/13	15.38
Patient 4	45/46	97.83
Patient 5	2/13	15.38

The decision of using a random selection of proteins to generate the EDAS is essential. So that population difference is covered. Lifestyle habits and behaviors affect human general health, like cigarette smoking, excessive alcohol consumption, excessive sunlight exposure, poor diet, lack of exercise, medical drugs, change of hormones, radiation, viruses, bacteria, and environmental chemicals. Chemical factors might be in the air, water, food, and/or workplace. The genetic makeup is essential so that these mentioned factors can lead to malignant transformation (American Cancer Society, 2017; Fymat, 2017; Iqbal, 2017; Ellberg et al., 2018; Ukawa et al., 2018). Because of the complicated interplay of many habits and behaviors, it is difficult to predict which combination of these habits and behaviors is accountable for certain cancer. The cause of cancer is still unknown and the human body's readiness to be diseased is unpredictable.

One of the important areas of research today is attempting to identify the association between the habits and behavior of an individual and diseases, specifically, Malignant Tumor. From this point, this EDAS can be used to find the association between normal proteins (environmental factors) and diseases that are difficult to diagnose and propose justifications for these diseases (further research). However, this model does not cover case prognosis, i.e., malignancy staging or infection severity.

## Conclusion

This paper is focused on issues related to the design and implementation of advanced technology based on using mass spectrometry in clinical practice. Its main purpose is to help in diagnosing disease conditions in the early stages precisely. The technique in this stage is based on hypothetical generated data. The technique is tested by generating databases each with 100,000 cases covering 20 pathogens and 20 malignancies. The technique conducted counts on random cases generation. In the future, the database will be generated from real patients. Consequently, the same code can be applied to discover biomarkers. Also, we will attempt to find the association between normal proteins and diseases by using association mining rule algorithms. Finally, discovering unique protein(s) associated with a particular disease can be used to develop vaccines which will be a very interesting future direction.

The presented diagnostic model can be used in clinical laboratories. In real life, the application can be initiated by some cases (normal and abnormal) and then incremented during its lifetime. The set of biomarkers of a particular disease will be built incrementally by adding new cases. By the time the set of biomarkers of a specific disease will be stable. The stability of the biomarker set of a particular disease is the indicator of knowledge completeness for this disease. In effect; the tool can be trusted for diagnosis of a disease if its biomarker set is stable. Clinician and Biologists will be the main users of the tool.

## Author Contributions

RE is a Ph.D student working on her thesis topic on disease diagnosis, where MR owns the patent of discovering Erythrocytes Dynamic Antigens Store and all the authors working on applying it and developing ML methodology to predict disease proteins (disease profiling).

### Conflict of Interest Statement

The authors declare that the research was conducted in the absence of any commercial or financial relationships that could be construed as a potential conflict of interest.

## Footnotes

^1^http://www.biomarkersconsortium.org

^2^Biomarkers in Risk Assessment: Validity and Validation, Environmental Health Criteria 222, WHO

^3^http://www.cancer.gov/dictionary/?CdrID=45618

^4^Biomarkers in Risk Assessment: Validity and Validation, Environmental Health Criteria 222, WHO
